# Antihyperalgesic Activities of Endocannabinoids in a Mouse Model of Antiretroviral-Induced Neuropathic Pain

**DOI:** 10.3389/fphar.2017.00136

**Published:** 2017-03-20

**Authors:** Neha Munawar, Mabayoje A. Oriowo, Willias Masocha

**Affiliations:** ^1^Department of Pharmacology and Toxicology, Faculty of Medicine, Kuwait UniversitySafat, Kuwait; ^2^Department of Pharmacology and Therapeutics, Faculty of Pharmacy, Kuwait UniversitySafat, Kuwait

**Keywords:** endocannabinoid, 2-arachidonoyl glycerol, anandamide, neuropathic pain, hyperalgesia, ddC, nucleoside reverse transcriptase inhibitor, antiretroviral

## Abstract

**Background:** Nucleoside reverse transcriptase inhibitors (NRTIs) are the cornerstone of the antiretroviral therapy for human immunodeficiency virus/acquired immune deficiency syndrome (HIV/AIDS). However, their use is sometimes limited by the development of a painful sensory neuropathy, which does not respond well to drugs. Smoked cannabis has been reported in clinical trials to have efficacy in relieving painful HIV-associated sensory neuropathy.

**Objectives:** The aim of this study was to evaluate whether the expression of endocannabinoid system molecules is altered during NRTI-induced painful neuropathy, and also whether endocannabinoids can attenuate NRTI-induced painful neuropathy.

**Methods:** BALB/c mice were treated with 25 mg/kg of 2′,3′-dideoxycytidine (ddC, zalcitabine), a NRTI, to induce thermal hyperalgesia. The expression of endocannabinoid system molecules was evaluated by real time polymerase chain reaction in the brain, spinal cord and paw skin at 6 days post ddC administration, a time point when mice had developed thermal hyperalgesia. The effects of the endocannabinoids, *N*-arachidonoyl ethanolamine (AEA) and 2-arachidonoyl glycerol (2-AG), the cannabinoid type 1 (CB1) receptor antagonist AM 251, CB2 receptor antagonist AM 630, and G protein-coupled receptor 55 (GPR55) antagonists ML193 and CID 16020046 on ddC-induced thermal hyperalgesia were evaluated using the hot plate test.

**Results:** ddC treatment resulted in thermal hyperalgesia and increased transcripts of the synthesizing enzyme *Plcβ1* and decreased *Daglβ* in the paw skins, but not *Napepld*, and *Daglα* compared to vehicle treatment. Transcripts of the inactivating enzymes *Faah* and *Mgll* were downregulated in the brain and/or paw skin but not in the spinal cord of ddC-treated mice. Both AEA and 2-AG had antihyperalgesic effects in mice with ddC-induced thermal hyperalgesia, but had no effect in ddC-naïve mice. The antihyperalgesic activity of AEA was antagonized by AM251 and AM630, whereas the activity of 2-AG was antagonized by AM251, ML193 and CID 16020046, but not by AM630.

**Conclusion:** These data show that ddC induces thermal hyperalgesia, which is associated with dysregulation of the mRNA expression of some endocannabinoid system molecules. The endocannabinoids AEA and 2-AG have antihyperalgesic activity, which is dependent on cannabinoid receptor and GPR55 activation. Thus, agonists of cannabinoid receptors and GPR55 could be useful therapeutic agents for the management of NRTI-induced painful sensory neuropathy.

## Introduction

Pain is a major cause of poor quality of life among human immunodeficiency virus (HIV) infected patients (Larue et al., 1997). It can be due to the HIV infection, opportunistic infections, cancers and drug treatment (Nair et al., 2009). Pain is almost underestimated as it is subjective especially in HIV/acquired immune deficiency syndrome (AIDS) patients where the main focus is on other principal symptoms of the infection such as immunosuppression and opportunistic infections (Larue et al., 1997; Ferrari et al., 2006). About 25–50% of all pain clinic visits are due to neuropathic pain (Verma et al., 2004). Neuropathic pain is felt by around 20–40% of people with AIDS (Hitchcock et al., 2008; Ebirim and Otokwala, 2013). Some of the symptoms of neuropathic pain include hyperalgesia (an increased response to normally painful stimuli), allodynia (pain triggered by normally non-painful stimuli, such as cloth rubbing against the skin) and spontaneous sensations such as burning, shooting, numbness, spasm and prickling (Dworkin et al., 2003; Cherry et al., 2012). It particularly affects the feet, hands and face, thus it can make the performance of day to day tasks such as cooking and other physical tasks very difficult (Hitchcock et al., 2008). This can have a serious negative impact on psychosocial well-being and overall quality of life of patients. These symptoms might also lead to discontinuation of antiretroviral therapy resulting in failure to suppress viral replication and worsening of HIV infection/AIDS.

Nucleoside reverse transcriptase inhibitors (NRTIs), in combination with other antiretroviral drugs, are effective in controlling the replication of HIV and form the backbone of most regimens used in the treatment of HIV/AIDS. However, their use is sometimes hampered by adverse effects including the development of dose-dependent painful peripheral neuropathy. Some NRTIs, such as didanosine (ddI), stavudine (d4T), and zalcitabine (ddC), have been removed/are being removed from regimens because of the development of peripheral neuropathy in about 15–30% of patients (Fichtenbaum et al., 1995; Moyle and Sadler, 1998; Dalakas, 2001). First-line medications recommended for managing neuropathic pain include amitriptyline, nortriptyline, duloxetine, venlafaxine, gabapentin, pregabalin, and 5% topical lidocaine (Dworkin et al., 2007). However, patients are not satisfied with current treatment options because of inadequate pain relief (Shlay et al., 1998; Simpson et al., 2000; Phillips et al., 2010) and the adverse side effect profiles which limit therapeutic efficacy and contribute to poor pain relief (Rahn and Hohmann, 2009). However, some HIV patients with painful neuropathy report relief after using cannabis (Woolridge et al., 2005; Phillips et al., 2010). Smoked cannabis has been reported in two randomized clinical trials to have efficacy in relieving painful HIV-associated sensory neuropathy (Abrams et al., 2007; Ellis et al., 2009). It has been observed that treatment with cannabinoid receptor agonists such as WIN 55,212-2 produced antinociception and antihyperalgesia in rodent models of HIV and NRTI-induced neuropathic pain (Wallace et al., 2007a,b). However, neither changes in the endocannabinoid system nor the effects of endocannabinoids against NRTI-induced painful neuropathy have been investigated.

Endocannabinoids and exogenous cannabinoid ligands produce their effects via two known cannabinoid receptors, CB1 and CB2 receptors (Guindon and Hohmann, 2009). Endogenous cannabinoid ligands are lipid molecules that are produced from phospholipid precursors in the cell membrane (Di Marzo et al., 1998; Piomelli, 2005) upon an “on demand” fashion (Giuffrida et al., 1999; Varma et al., 2001; Kim et al., 2002). *N*-arachidonoyl-ethanolamine (anandamide, AEA) and 2-arachidonoyl-glycerol (2-AG) are the two most extensively studied endocannabinoids so far (Di Marzo and Piscitelli, 2015). AEA is a partial agonist at the CB1 and CB2 receptors (Hillard et al., 1999; Howlett et al., 2002; Chavez et al., 2010; Grueter et al., 2010). On the other hand, 2-AG behaves as a complete agonist at CB1 and 2 receptors (Stella et al., 1997; Gonsiorek et al., 2000; Savinainen et al., 2001; Howlett et al., 2002). Several pathways are involved in the synthesis of AEA and 2-AG (Di Marzo, 2008). *N*-acylphosphatidyl ethanolamine specific phospholipase D (NAPE-PLD) hydrolyses *N-*arachidonylphosphatidyl ethanolamine (NAPE) to produce AEA and phosphatidic acid (Natarajan et al., 1981; Schmid et al., 1983; Di Marzo et al., 1994; Okamoto et al., 2004). 2-AG is synthesized through the catalysis of the membrane bound phosphatidylinositol-4,5-biphosphate (PIP2) to diacylglycerol (DAG) by phospholipase C (PLC)-β1 (Farooqui et al., 1989; Ayakannu et al., 2013; Di Marzo and Piscitelli, 2015). The intermediate DAG is further catalyzed by the action of one of two diacylglycerol lipases (DAGLs), DAGL-α and DAGL-β, to 2-AG (Bisogno et al., 2003; Tanimura et al., 2010). In order to maintain endocannabinoid homeostasis, AEA and 2-AG after release in the synaptic space are either transported back into the cells by a transporter or degraded by enzymes. AEA is hydrolyzed to arachidonic acid and ethanolamine by fatty acid amide hydrolase (FAAH), an intracellular membrane-bound enzyme (Cravatt et al., 1996). 2-AG is hydrolyzed to arachidonic acid and glycerol by the enzyme monoacylglycerol lipase (MAGL) (Dinh et al., 2002; Saario et al., 2004).

The objective of this study was to evaluate whether the expression of molecules of the endocannabinoid system is altered in the central nervous system (CNS) and the periphery of mice during NRTI-induced painful neuropathy, and also whether endocannabinoids can attenuate NRTI-induced painful neuropathy.

## Materials and Methods

### Animals

The animals used in this study were female BALB/c mice (2–3 months old; 20–25 g) supplied by the Animal Resources Centre at the Health Sciences Centre (HSC), Kuwait University, Kuwait. The mice were kept in temperature controlled (24 ± 1°C) rooms with food and water *ad libitum*. All experiments were performed during the same period of the day (8:00 AM to 4:00 PM) to exclude diurnal variations in pharmacological effects. The animals were handled in compliance with Directive 2010/63/EU of the European Parliament and of the Council on the protection of animals used for scientific purposes. All animal experiments were approved by the Ethical Committee for the use of Laboratory Animals in Teaching and in Research, HSC, Kuwait University.

### Administration of 2′,3′-Dideoxycytidine (ddC) to Induce Neuropathic Pain

2***′***,3′-dideoxycytidine (ddC, zalcitabine) (Sigma-Aldrich, St. Louis, MO, USA) was prepared freshly in normal saline (0.9% NaCl) on the day of the experiment. ddC 25 mg/kg or its vehicle was administered to mice in a single intraperitoneal (i.p.) injection, in a volume of 10 ml/kg. This treatment regimen has been reported to produce painful neuropathy in mice (Sanna et al., 2014).

### Drug Administration

All the drugs were purchased from Tocris, Bristol, UK.*N-*arachidonoyl ethanolamine (AEA, anandamide), was dissolved in Tocrisolve; 2-arachidonoyl glycerol (2-AG) in normal saline containing 5% ethanol, 5% cremophor, and 5% DMSO (Guindon et al., 2011); AM 251 and AM 630 in normal saline containing 5% Tween 80 and 5% propylene glycol; ML193 and CID 16020046 in normal saline containing 5% ethanol, 5% cremophor, and 5% DMSO. The drugs and their vehicles were freshly prepared and further diluted with normal saline to lower concentrations before administration and administered i.p. to mice at a volume of 10 ml/kg body mass.

The doses of AEA and 2-AG, 1, 10, and 20 mg/kg, were chosen based on those previously shown to have antinociceptive and/or antihyperalgesic activity in mice (Mechoulam et al., 1995; Calignano et al., 2001). The drugs were administered to naïve mice and ddC-treated mice at 6 days after administration of ddC, when mice had developed thermal hyperalgesia.

To evaluate the receptors involved in the antihyperalgesic activities of the endocannabinoids CB1 (AM 251 3 mg/kg), CB2 (AM 630 3 mg/kg), and GPR55 (ML193 and CID 16020046 both at 10 mg/kg) antagonists were administered 15 min before the administration of AEA and 2-AG to mice with ddC-induced thermal hyperalgesia.

### Assessment of Thermal Nociception

Reaction latencies of mice to hot plate (Panlab SL, Barcelona, Spain) at 55 ± 1°C in the form of the first sign of nociception, paw licking, flinching or jump response to avoid the heat were measured, as described before (Parvathy and Masocha, 2013), before (baseline latency), at day 6 after injection of ddC and at various times after drug treatment. A cut-off period of 20 s was maintained to avoid damage to the paws.

### Tissue Preparation and Real Time RT-PCR

Mice were anesthetized with halothane, sacrificed by decapitation on day 6 post-administration of ddC. Brains, spinal cords and paw skins were dissected, snap frozen in liquid nitrogen and kept at - 70°C prior to mRNA extraction.

Gene transcripts of cannabinoid type 1 receptor (*Cnr1*), *Cnr2, N-*arachidonoyl ethanolamine-specific phospholipase D (*Napepld*), phospholipase C-beta 1 (*Plcβ1*), diacylglycerol lipase-alpha (*Daglα*), *Daglβ*, fatty acid amide hydrolase (*Faah*), and monoacylglycerol lipase (*Mgll*) were quantified in the brains, spinal cords and paw skins of vehicle-treated and ddC-treated mice by real time polymerase chain reaction (PCR). Total RNA was extracted from the fresh frozen brains, spinal cords and paw skins using the RNeasy Kit (Qiagen GmbH), reverse-transcribed, and the mRNA levels were quantified on an ABI Prism^®^ 7500 sequence detection system (Applied Biosystems) as previously described (Masocha, 2009). The primer sequences which were used, listed in **Table 1**, were ordered from Invitrogen (Life Technologies) and/or synthesized at the Research Core Facility (RCF), HSC, Kuwait University. Threshold cycle (Ct) values for all cDNA samples were obtained and the amount of mRNA of individual animal sample (*n* = 5–10 per group) was normalized to *Ppia* (cyclophilin A, housekeeping gene) (ΔCt). The relative amount of target gene transcripts was calculated using the 2^-ΔΔCt^ method as described previously (Livak and Schmittgen, 2001). These values were then used to calculate the mean and standard error of mean of the relative expression of the target gene mRNA in the brains, spinal cords and paw skins of vehicle- and ddC-treated mice.

**Table 1:** Polymerase chain reaction (PCR) primer sequences of cyclophilin A and endocannabinoid system molecules.

Gene	Polarity	
	Sense	Anti-sense
	Sequence 5′–3′	Sequence 5′–3′
*Ppia* (cyclophilin A)	GCTTTTCGCCGCTTGCT	CTCGTCATCGGCCGTGAT
*Cnr1* (CB1 receptor)	GTTCTGATCCTGGTGGTGTTG	GTTCAGCAGGCAGAGCATAC
*Cnr2* (CB2 receptor)	TCTGGAAAGCCCACCGGCATGTAG	CAAGGCACAGCATGGAACAGAAGG
*Daglα*	GTCCTGCCAGCTATCTTCCTC	CGTGTGGGTTATAGACCAAGC
*Daglβ*	AGCGACGACTTGGTGTTCC	GCTGAGCAAGACTCCACCG
*Faah*	GCTGTGCTCTTTACCTACCTG	GAAGCATTCCTTGAGGCTCAC
*Mgll*	TGATTTCACCTCTGGTCCTTG	GTCAACCTCCGACTTGTTCC
*Napepld*	GGGCGGCTCTCACTTTCTA	ACACTTGTGCTTATAGGTCATTTAAT
*Plcβ1*	GCCCCTGGAGATTCTGGAGT	GGGAGACTTGAGGTTCACCTTT

### Statistical Analyses

Statistical analyses were performed using unpaired Student’s *t*-test (to compare the effects of ddC to those of the vehicle on mRNA expression on day 6 post ddC injection; and to compare the effects of ddC on reaction latency on day 6 post-injection and pretreatment reaction latency), one-way analysis of variance (ANOVA) followed by Bonferroni’s multiple comparison post-tests (to compare the effects of pretreatment with CB receptor and GPR55 antagonists before administration of either AEA or 2-AG to the antihyperalgesic effects of AEA and 2-AG in mice with ddC-induced thermal hyperalgesia at day 6 post-injection of ddC), or two-way repeated measures ANOVA followed by Bonferroni’s multiple comparison post-tests (to compare the effects of ddC to vehicle on reaction latency over time; and to compare the effects of AEA and 2-AG to that of the vehicle in mice with ddC-induced thermal hyperalgesia) using GraphPad Prism software (version 5.0). The differences were considered significant at *p* < 0.05. The results in the text and figures are expressed as the means ± SEM.

## Results

### ddC-Induced Thermal Hyperalgesia in Female BALB/c Mice

**FIGURE 1:**
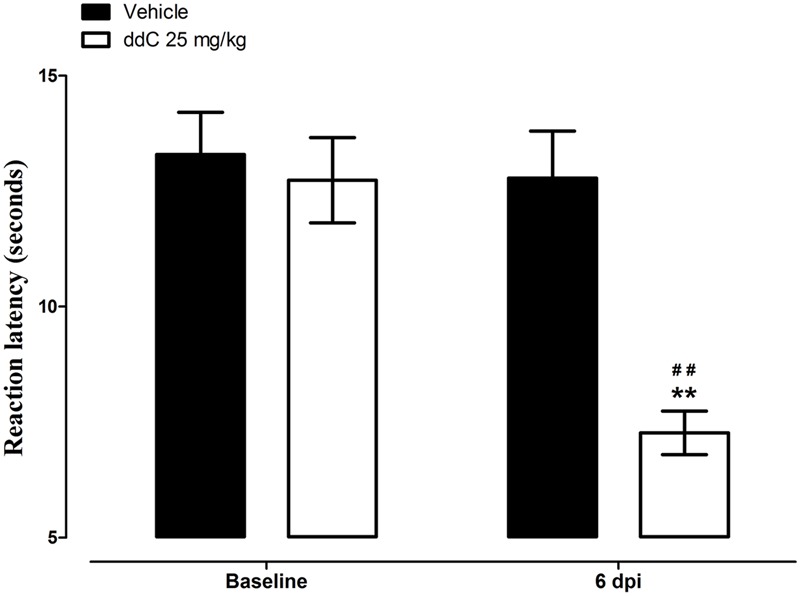
**2′,3′-Dideoxycytidine (ddC)-induced thermal hyperalgesia in female BALB/c mice.** Reaction latency of mice before treatment and at day 6 post-injection (6 dpi) of ddC in a hot plate test. Each bar represents the mean ± SEM of values obtained from 18 animals. ^∗∗^*p* < 0.01 compared to drug vehicle at the same day after treatment (two-way repeated measures ANOVA followed by Bonferroni’s Multiple Comparison Test) and ^##^*p* < 0.01 compared to pretreatment baseline values (Student’s *t*-test).

Mice treated with ddC developed thermal hyperalgesia on day 6 after treatment, i.e., reduction in reaction latency compared to the baseline latency and vehicle-treated mice (7.3 ± 0.5 s compared to 12.7 ± 0.9 s and 12.8 ± 1.0 s, respectively; *n* = 18 for both vehicle- and ddC-treated mice; *p* < 0.001 for both comparisons; **Figure 1**). There was a significant interaction between treatment and time after treatment with ddC (*F*_1,34_ = 60.72, *p* < 0.0001).

The mRNA expression of endocannabinoid molecules (in the brain, spinal cord, and paw skin) and the antihyperalgesic activity the endocannabinoids AEA and 2-AG were analyzed at day 6 in separate groups of mice, at this time point mice showed ddC-induced thermal hyperalgesia.

### Expression of the Endocannabinoid System Molecules mRNA during ddC-Induced Thermal Hyperalgesia in the Brain, Spinal Cord, and Paw Skin

**FIGURE 2:**
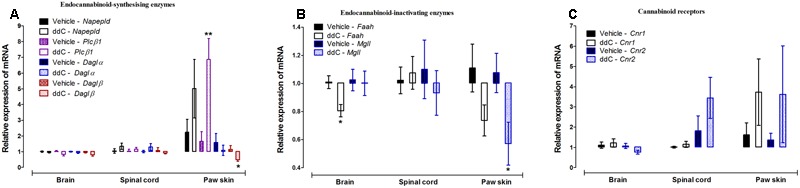
**Effects of ddC on endocannabinoid system molecules transcript levels in the brain, spinal cord and paw skin of female BALB/c mice.** Relative mRNA expression of **(A)** endocannabinoid-synthesizing enzymes *Napepld, Plcβ1, Daglα* and *Daglβ*, **(B)** endocannabinoid-inactivating enzymes *Faah* and *Mgll*, and **(C)** cannabinoid receptors *Cnr1* and *Cnr2* in the brains, spinal cords, and paw skins of BALB/c mice on day 6 after administration of the ddC or its vehicle. Each bar represents the mean ± SEM of the values obtained from 6 to 10 vehicle-treated mice and 5–8 ddC-treated mice. ^∗^*p* < 0.05, ^∗∗^*p* < 0.01 compared to vehicle-treated mice (Student’s *t*-test).

Treatment with ddC did not affect the expression of endocannabinoid-synthesizing enzymes (*Napepld, Plcβ1, Daglα*, and *Daglβ*) in the brain or the spinal cord compared to vehicle treatment. However, treatment with ddC significantly increased the transcripts of *Plcβ1* (*p* = 0.0021), decreased the transcripts of *Daglβ* (*p* = 0.0406), but did not significantly affect the expression of *Daglα* and *Napepld* (*p* > 0.05) in the paw skin compared to vehicle treatment (**Figure 2A**).

Treatment with ddC did not affect the expression of endocannabinoid-inactivating enzymes (*Faah* and *Mgll*) in the spinal cord compared to vehicle treatment. However, treatment with ddC significantly decreased the transcripts of *Faah* (*p* = 0.0055), but did not significantly affect the expression of *Mgll* (*p* > 0.05) in the brain compared to vehicle treatment. In the paw skin, treatment with ddC significantly decreased the transcripts of *Mgll* (*p* = 0.0275), but did not significantly affect the expression of *Faah* (*p* > 0.05; **Figure 2B**).

The expression of the cannabinoid receptors *Cnr1* and *Cnr2* were not significantly modulated by treatment with ddC in all the three tissues analyzed, brain, spinal cord and paw skin, compared to vehicle treatment (*p* > 0.05; **Figure 2C**).

### Effects of Treatment with the Endocannabinoids AEA and 2-AG on Naïve Mice and Mice with ddC-Induced Thermal Hyperalgesia

Mice with ddC-induced thermal hyperalgesia and naïve mice were treated with 1, 10, and 20 mg/kg of the endocannabinoids AEA and 2-AG.

**FIGURE 3:**
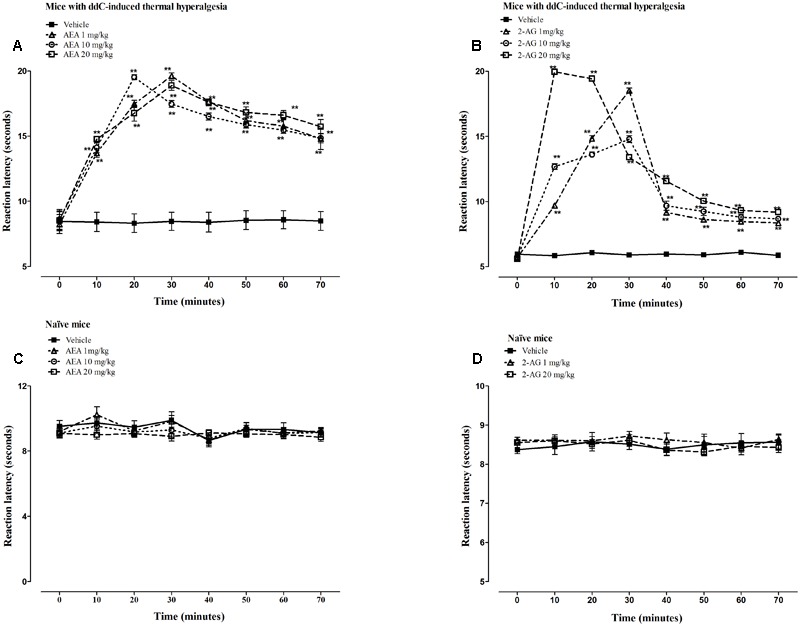
**Antihyperalgesic effects of AEA and 2-AG in female BALB/c mice with ddC-induced thermal hyperalgesia.** Reaction latency times at day 6 post-injection of ddC at different times after treatment with **(A)** AEA (1, 10, and 20 mg/kg) or its vehicle, and **(B)** 2-AG (1, 10, and 20 mg/kg) or its vehicle in a hot-plate test. Reaction latency times of naïve mice at different times after treatment with **(C)** AEA (1, 10, and 20 mg/kg) or its vehicle, and **(D)** 2-AG (1 and 20 mg/kg) or its vehicle in a hot-plate test. Each bar represents the mean ± SEM of values obtained from 8 to 9 animals. ^∗∗^*p* < 0.01 compared to drug vehicle at the same time point after treatment (two-way repeated measures ANOVA followed by Bonferroni’s Multiple Comparison Test).

The intraperitoneal administration of vehicle did not change the reaction latency to thermal stimuli in mice with ddC-induced thermal hyperalgesia compared to before administration at day 6 (*p* > 0.05; **Figures 3A,B**). However, all the doses (1, 10, and 20 mg/kg) of AEA and 2-AG administered produced significant increase in reaction latency in mice with ddC-induced thermal hyperalgesia at all time points from 10 to 70 min post-drug administration, when the experiment was terminated, compared to mice treated with vehicle and before endocannabinoid administration at day 6 (*p* < 0.01; **Figures 3A,B**). There was a significant interaction between treatment and time after treatment for AEA doses of 1 mg/kg (*F*_7,98_ = 50.22, *p* < 0.0001), 10 mg/kg (*F*_7,98_ = 73.17, *p* < 0.0001), and 20 mg/kg (*F*_7,98_ = 48.48, *p* < 0.0001) in mice with ddC-induced thermal hyperalgesia. There was also a significant interaction between treatment and time after treatment for 2-AG doses of 1 mg/kg (*F*_7,98_ = 662.69, *p* < 0.0001), 10 mg/kg (*F*_7,98_ = 218.53, *p* < 0.0001) and 20 mg/kg (*F*_7,98_ = 1230.23, *p* < 0.0001) in mice with ddC-induced thermal hyperalgesia.

The intraperitoneal administration of AEA or 2-AG did not change the reaction latency to thermal stimuli in naïve mice (without ddC treatment) at any time point compared to mice treated with vehicle and before endocannabinoid administration (*p* > 0.05; **Figures 3C,D**). There was no significant interaction between treatment and time after treatment for AEA doses of 1 mg/kg (*F*_7,105_ = 0.55, *p* = 0.7928) and 10 mg/kg (*F*_7,105_ = 0.48, *p* = 0.8456), but at 20 mg/kg (*F*_7,105_ = 2.61, *p* = 0.0155) in mice with ddC-induced thermal hyperalgesia. There was also no significant interaction between treatment and time after treatment for 2-AG doses of 1 mg/kg (*F*_7,98_ = 0.79, *p* = 0.5996), and 20 mg/kg (*F*_7,98_ = 1.07, *p* = 0.3871) in naïve mice.

### Effects of Cannabinoid Receptor and GPR55 Antagonists on the Antihyperalgesic Activities of the Endocannabinoids AEA and 2-AG in Mice with ddC-Induced Thermal Hyperalgesia

**FIGURE 4:**
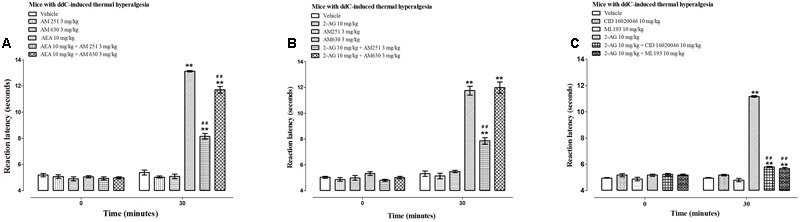
**Effects of CB receptor and GPR55 antagonists on the antihyperalgesic effects of AEA and 2-AG in female BALB/c mice with ddC-induced thermal hyperalgesia at day 6 post-injection of ddC.** Effects of **(A)** a CB1 receptor antagonist AM 251 and a CB2 receptor antagonist AM 630 on the antihyperalgesic effects of AEA, **(B)** a CB1 receptor antagonist AM 251 and a CB2 receptor antagonist AM 630 on the antihyperalgesic effects of 2-AG, and **(C)** GPR55 antagonists CID 16020046 and ML193 on the antihyperalgesic effects of 2-AG in BALB/c mice with ddC-induced thermal hyperalgesia 30 min after administration in the hot plate test. Each bar represents the mean ± SEM of values obtained from 8 to 16 animals.^∗∗^*p* < 0.01 compared to drug vehicle at the same time point after treatment and ^##^*p* < 0.01 to mice treated with AEA or 2-AG at the same time point after treatment (one-way ANOVA followed by Bonferroni’s Multiple Comparison Test).

The administration of the CB1 receptor antagonist AM 251 (3 mg/kg) or the CB2 antagonist AM 630 (3 mg/kg) to mice with ddC-induced thermal hyperalgesia did not alter the reaction latency to hot-plate test compared to vehicle-treated mice at 30 min post-treatment (*p* > 0.05; **Figure 4**).

The CB1 receptor antagonist AM 251 significantly antagonized the antihyperalgesic effect of both AEA and 2-AG, i.e., a 38% reduction in reaction latency to AEA, 13.1 ± 0.1 s for AEA alone compared to 8.2 ± 0.2 s for AEA + AM 251 and 33% reduction in reaction latency to 2-AG, 11.7 ± 0.3 s for 2-AG compared to 7.9 ± 0.3 s for 2-AG + AM 251 (*p* < 0.01; **Figures 4A,B**).

The CB2 receptor antagonist AM 630 significantly antagonized the antihyperalgesic effect of AEA but not 2-AG, i.e., a 11% reduction in reaction latency to AEA, 13.1 ± 0.1 s for AEA alone compared to 11.7 ± 0.3 s for AEA + AM 630 (*p* < 0.01; **Figure 4A**) and no difference in reaction latency to 2-AG, 11.7 ± 0.3 s for 2-AG compared to 12.0 ± 0.4 s for 2-AG + AM 630 (*p* > 0.05; **Figure 4B**).

Since the antihyperalgesic activity of 2-AG was partially antagonized by the CB1 receptor antagonist but was not affected by the CB2 receptor antagonist, and 2-AG has been reported to activate GPR55 (Ryberg et al., 2007), the effects of two GPR55 antagonists CID 16020046 and ML193 on the activities of 2-AG were also evaluated. The administration of the GPR55 antagonists CID 16020046 (10 mg/kg) and ML193 (10 mg/kg) to mice with ddC-induced thermal hyperalgesia did not alter the reaction latency to hot-plate test compared to vehicle-treated mice at 30 min post-treatment (*p* > 0.05; **Figure 4C**). The GPR55 antagonists CID 16020046 and ML193 significantly antagonized the antihyperalgesic effect of 2-AG, i.e., a 48% reduction in reaction latency to 2-AG caused by pretreatment with CID 16020046, 11.2 ± 0.1 s for 2-AG alone compared to 5.8 ± 0.04 s for 2-AG + CID 16020046 and 49% reduction in reaction latency to 2-AG caused by pretreatment with ML193, 11.2 ± 0.1 s for 2-AG alone compared to 5.7 ± 0.1 s for 2-AG + ML193 (*p* < 0.01; **Figure 4C**).

## Discussion

This study presents the first data on antihyperalgesic activities of endocannabinoids and the expression of endocannabinoid molecules mRNA in the CNS and periphery during NRTI-induced thermal hyperalgesia. Mice with ddC-induced thermal hyperalgesia had altered mRNA expression of endocannabinoid-synthesizing enzymes *Plcβ1* and *Daglβ* in the paw skin, and endocannabinoid-inactivating enzymes *Faah* and *Mgll* in the brain and paw skin, respectively. The endocannabinoids AEA and 2-AG had antihyperalgesic activity against ddC-induced thermal hyperalgesia but had no activity in naïve mice. The antihyperalgesic activity of AEA was dependent on activation of both CB1 and CB2 receptors, whereas that of 2-AG was dependent on CB1 receptor and GPR55, but not CB2 receptor.

Changes in endocannabinoid expression have been found in various models of neuropathic pain (Jhaveri et al., 2007). In the periphery, AEA has been found to be increased in the paw skin and dorsal root ganglia (DRG) of rats with spinal nerve ligation (SNL)-induced painful neuropathy (Mitrirattanakul et al., 2006; Jhaveri et al., 2007). 2-AG has been found to be increased in the DRG of rats with SNL-induced painful neuropathy (Mitrirattanakul et al., 2006). In the CNS, AEA, and/or 2-AG have been reported to be increased in different areas of the brain and spinal cord of rats with neuropathic pain induced by chronic constriction injury (CCI) of the sciatic nerve (Palazzo et al., 2006; Petrosino et al., 2007). In an animal model of chemotherapy-induced neuropathic pain (CINP) AEA and 2-AG were increased in the spinal cord, whilst 2-AG, but not AEA, was decreased in the paw skin (Guindon et al., 2013). Endocannabinoid molecules, such as AEA and 2-AG, are synthesized in an on demand fashion (Luchicchi and Pistis, 2012). Thus, changes in the enzymes that synthesize or degrade them would have a significant effect on the amount of endocannabinoids available when needed. There were no changes in the expression of mRNA of *Napepld*, the main AEA synthesizing enzyme, in DRGs or in the spinal cord of rats with CCI-induced neuropathic pain (Malek et al., 2014). However, NAPE-PLD immunoreactivity was decreased in the DRGs of rats with SNL (Sousa-Valente et al., 2016). In the current study, ddC-induced neuropathic pain did not significantly affect the level of the mRNA of *Napepld* in the CNS or periphery, similar to what has been found in the CCI model (Malek et al., 2014). However, there was an increase in the mRNA expression of *Plcβ1*, an enzyme involved in the synthesis of DAG, an intermediate in the synthesis of 2-AG, in mice with ddC-induced neuropathic pain in the periphery but not in the CNS. On the other hand, there were no changes in the expression of mRNA of *Plcβ1* mRNA in DRGs or in the spinal cord of rats with CCI-induced neuropathic pain (Malek et al., 2014). The protein levels of DAGL-α, one of the main enzymes involved in the synthesis of 2-AG from DAG, were decreased in the spinal cord of mice with diabetic neuropathic pain (Ikeda et al., 2013). In the current study, the mRNA expression of *Daglβ*, but not *Daglα*, was decreased in the paw skin but not spinal cord or brain of mice with ddC-induced neuropathic pain. The mRNA expression of *Faah*, the main enzyme in the degradation of AEA, was increased in the spinal cord but not in the DRGs (Malek et al., 2014). Similarly, in a rat model of CINP *Faah* was increased in the spinal cord but not in the paw skin (Guindon et al., 2013). FAAH immunoreactivity was increased in the DRGs of rats with SNL (Sousa-Valente et al., 2016). In the rostroventromedial medulla (RVM) area of the brain of rats with diabetic neuropathy FAAH protein levels were increased (Silva et al., 2016). In contrast, mRNA expression of *Faah* was decreased in the brain but not the spinal cord, and showed a tendency toward decrease in the paw skin of mice with ddC-induced thermal hyperalgesia. There were no changes in the mRNA expression of *Mgll*, the main enzyme in the degradation of 2-AG, in the spinal cord or paw skins of rats with CINP (Guindon et al., 2013). In contrast mRNA expression of *Mgll* was decreased in the paw skin, but not spinal cord and brain, of mice with ddC-induced thermal hyperalgesia. The findings of the current study show a unique characteristic of a decrease in the mRNA expression of the enzymes that inactivate AEA and 2-AG, which might result in low levels of the enzymes and increased endocannabinoids.

*N*-arachidonoyl ethanolamine and 2-AG are important endogenous inhibitors of nociception (Rodriguez de Fonseca et al., 2005; Guindon and Hohmann, 2009). Exogenous administration locally in the paw skin of both AEA and 2-AG in rats with partial sciatic nerve ligation (PNL)- or diabetes-induced neuropathic pain and intrathecally into the spinal cord of rats with CCI-induced neuropathic pain have produced antihyperalgesic activities (Guindon and Beaulieu, 2006; Desroches et al., 2008; Schreiber et al., 2012; Starowicz et al., 2012; Desroches et al., 2014). In the current study, systemic administration (i.p.) of both AEA and 2-AG produced antihyperalgesic activities and abrogated ddC-induced thermal hyperalgesia, but had no activity in naïve mice (mice without neuropathic pain). Calignano et al. (1998) observed that locally administered AEA had antinociceptive effects, whereas intraperitoneally administered AEA had no effect on nociception in naïve mice. However, in another study they observed that intraperitoneally administered AEA had antinociceptive effects that lasted up to 20 min (Calignano et al., 2001). In another study, AEA administered intraperitoneally did not produce antinociceptive effects in rats (Costa et al., 1999). Our findings of antihyperalgesic activity of AEA and 2-AG administered intraperitoneally, that lasted up to 70 min when the experiment was terminated, in mice with ddC-induced thermal hyperalgesia but not in naïve mice suggest that possibly the reduction in the inactivating enzymes FAAH and MAGL in mice with ddC-induced thermal hyperalgesia contributed to the prolonged antihyperalgesic activity of the endocannabinoids. This is in line with a study that showed that administration of AEA to mice that lack FAAH (FAAH^-/-^ mice) produced antinociception but AEA did not have antinociceptive effects in wild-type (FAAH^+/+^ mice) (Cravatt et al., 2001).

*N*-arachidonoyl ethanolamine and 2-AG produce their effects via two known cannabinoid receptors, CB1 and CB2 receptors (Guindon and Hohmann, 2009). AEA has been reported to produce its antinociceptive effects via activation of both CB1 and CB2 receptors (Schreiber et al., 2012). However, other studies have shown that AEA produces its antinociceptive effects via activation of CB1 receptors but not CB2 receptors (Calignano et al., 1998; Guindon and Beaulieu, 2006). Pre-treatment with either a CB1 or CB2 antagonist antagonized the antihyperalgesic activity of AEA in mice with ddC-induced thermal hyperalgesia. The antagonism was much more with the CB1 antagonist than the CB2 antagonist, thus suggesting that AEA had antihyperalgesic activity mainly via CB1 receptors although CB2 receptors also had a minor role. Pre-treatment with a CB1 antagonist, but not a CB2 antagonist, antagonized the antihyperalgesic activity of 2-AG in mice with ddC-induced thermal hyperalgesia. Thus, suggesting that 2-AG had antihyperalgesic activity via CB1 receptors but not CB2 receptors. This is in contrast with a study done in rats with PNL-induced neuropathic pain where both CB1 and CB2 antagonists equally inhibited the antihyperalgesic effects of 2-AG administered locally, subcutaneously in the dorsal surface of the hind paw (Desroches et al., 2008, 2014). The current study shows that CB1 receptors play a more important role than CB2 receptors in the antihyperalgesic effects of both endocannabinoids AEA and 2-AG against ddC-induced hyperalgesia.

Since the antihyperalgesic activity of 2-AG was partially antagonized by the CB1 receptor antagonist but was not affected by the CB2 receptor antagonist, and 2-AG has been reported to activate GPR55 (Ryberg et al., 2007), the effects of two GPR55 antagonists CID 16020046 and ML193 were also evaluated. We used two different antagonists of the same receptors because we did not find any reports of GPR55 receptor antagonists on the antinociceptive or antihyperalgesic activity of 2-AG. Pre-treatment with either GPR55 antagonist inhibited the antihyperalgesic activity of 2-AG in mice with ddC-induced thermal hyperalgesia much more than the CB1 receptor antagonist. Loss of GPR55 (GPR55^-/-^ mice) resulted in increased sensitivity to thermal nociception, thermal hyperalgesia (Staton et al., 2008; Wu et al., 2013; Bjursell et al., 2016). However, GPR55^-/-^ mice did not develop PNL-induced hyperalgesia, whereas the wild-type mice did, suggesting that GPR55 has a pro-nociceptive activity (Staton et al., 2008). Lysophosphatidylinositol (LPI), which is considered the main endogenous agonist of GPR55 (Oka et al., 2007; Henstridge et al., 2009), has pro-nociceptive activities (Deliu et al., 2015). Thus, our results show that 2-AG, in contrast to LPI, produces antinociceptive activity via activation of GPR55 receptors. Possibly 2-AG and LPI bind to and activate GPR55 at different sites or in different ways. This possibility warrants further research.

## Conclusion

Our results show that ddC induces thermal hyperalgesia that is associated with dysregulation of the mRNA expression of endocannabinoid molecules, more importantly downregulation of *Mgll* and *Faah*, which are involved in the inactivation of the endocannabinoids AEA and 2-AG. The endocannabinoids AEA and 2-AG had antihyperalgesic activity against ddC-induced thermal hyperalgesia, but had no activity in naïve mice, possibly due to the reduced mRNA expression of *Mgll* and *Faah* in mice with ddC-induced thermal hyperalgesia. The antihyperalgesic activity of AEA was dependent on activation of both CB1 and CB2 receptors, whereas that of 2-AG was dependent on CB1 receptors and GPR55, but not CB2 receptors. FAAH and MAGL inhibitors might not be very useful in the treatment of NRTI-induced neuropathic pain, since these two enzymes are reduced during ddC-induced thermal hyperalgesia. However, stable formulations of AEA and 2-AG or agonists of both CB1 receptors and GPR55, with activities similar to AEA and 2-AG, might be useful in the treatment of NRTI-induced neuropathic pain.

## Author Contributions

NM: performed the experiments, analyzed the data, wrote the paper; MO: contributed reagents/materials/analysis tools, analyzed the data, wrote the paper; WM: conceived and designed the experiments, contributed reagents/materials/analysis tools, analyzed the data, wrote the paper; all authors read and approved the final manuscript.

## Conflict of Interest Statement

The authors declare that the research was conducted in the absence of any commercial or financial relationships that could be construed as a potential conflict of interest.
